# A Dynamic Recommender System for Improved Web Usage Mining and CRM Using Swarm Intelligence

**DOI:** 10.1155/2015/193631

**Published:** 2015-07-01

**Authors:** Anna Alphy, S. Prabakaran

**Affiliations:** Department of Computer Science and Engineering, SRM University, Chennai 603203, India

## Abstract

In modern days, to enrich e-business, the websites are personalized for each user by understanding their interests and behavior. The main challenges of online usage data are information overload and their dynamic nature. In this paper, to address these issues, a WebBluegillRecom-annealing dynamic recommender system that uses web usage mining techniques in tandem with software agents developed for providing dynamic recommendations to users that can be used for customizing a website is proposed. The proposed WebBluegillRecom-annealing dynamic recommender uses swarm intelligence from the foraging behavior of a bluegill fish. It overcomes the information overload by handling dynamic behaviors of users. Our dynamic recommender system was compared against traditional collaborative filtering systems. The results show that the proposed system has higher precision, coverage, *F1* measure, and scalability than the traditional collaborative filtering systems. Moreover, the recommendations given by our system overcome the overspecialization problem by including variety in recommendations.

## 1. Introduction

The customer relationship management (CRM) entails the interaction of an organization with the current and future customers. The competitions in e-business require the efficient management of web usage data because a competitor's website may be only one click away. An improved understanding of customers' interest and their behaviors increases the profit of an organization. A personalized website in view of the customer's interests may bring customer's attention to the site more and thus increases the customer utility. The information regarding customer's interest and behavior also helps a website administrator to personalize or customize a web page for a user. Such increased usage of business websites online creates a huge amount of web usage information to manage causing information overload. To manage this information overload, efficient data mining techniques can be applied in addition to storing, retrieving, and managing these web usage data. These data mining techniques also may be used to identify the interesting patterns from web log data or online usage data.

The major challenges of online web usage data, in addition to information overloading, are its high dimensionality and dynamic nature caused by thousands of users. The online usage data is high dimensional because it contains huge number of clicks made by the users to purchase items. The online usage data represents the interest of human beings that are highly dynamic in nature. These dynamic behaviors may be due to the changes in the user's interest or due to the addition or deletion of web pages in a website. The personalization of the web for a user should also cope with these issues.

Designing and developing a suitable recommender system may be very much helpful in web personalization. It uses the recommendations provided by the recommender systems for providing the users with their items of interest. In the past many research works have been done in recommender systems. But most of the traditional recommender systems cannot handle the dynamic nature of online usage data.

Moreover the traditional recommender systems give limited recommendations. In traditional recommender systems, the number of iterations before convergence is high and also the quality of recommendations reduces with the increase in the number of users. The traditional recommender system also cannot balance the quality measures such as coverage and precision.

To overcome the above issues, we propose a WebBluegillRecom-annealing dynamic recommender system which could also provide recommendations to users. The proposed dynamic recommender system uses swarm intelligence approach. That is, in our dynamic recommender system, the recommendations are given not only based on users' interest but also based on the interest of the neighborhood users. Our dynamic recommender system also overcomes the overspecialization problem in many traditional recommender systems by providing variety in recommendations.

The performance of the proposed algorithm is compared with the traditional collaborative filtering recommender systems. The results of performance evaluation show that the proposed dynamic recommender system gives better predictions in less time without losing the quality in terms of coverage, precision, *F*1 measure, and scalability, compared with the traditional approaches. WebBluegillRecom-annealing recommender system overcomes the information overload and dynamic behavior challenges of many other recommendation systems.

The rest of the paper is organized as follows.  Section 2 describes the related works in the recommender systems.  Section 3 provides basic knowledge required.  Section 4 introduces the proposed WebBluegillRecom-annealing recommender system and Section 5 describes experimental results and performance evaluations.

## 2. Literature Survey

Ben Schafer et al. [1] introduced a collaborative filtering (CF) system that predicts a person's interest for an item by using that person's recorded interest with the recorded interest of a community of like-minded people. In the collaborative filtering approach, the neighbors of an active user *u* are defined as the users that are similar to *u* above a similarity threshold. Collaborative filtering supports group modeling. It gives recommendations based items liked by the users of the same interest. Dai et al. [2] introduced particle swarm chaos optimization mining algorithm (PSCOMA). It uses the strong global search ability of PSO and the strong local search ability of chaos optimization for the process of web usage mining. It offers a balancing between exploration and exploitation. Çelik et al. [3] introduced artificial bee colony data miner (ABC-Miner algorithm) for mining classification rules from large datasets. It uses intelligent foraging behavior of honey bees. Balabanovic [4] introduced Fab adaptive content based recommendation system. It gives web page recommendation service to users based on the recommendations of other users and also by analyzing their content. The main limitation of this approach is that since the recommendations are based only on users' previous ratings of items, users cannot explore new items other than the items mentioned in user profiles. The main advantage of this technique is that since group modeling is not used for recommendations brand new items can be easily added in the recommendations. Pazzani [5] recommends items to users based on demographic information about the users. Here the demographic attributes such as age, gender, and education can be used to classify users and predictions are given to a user based on this demographic information. Nasraoui and Petenes [6] presented a fuzzy interface engine that uses rules derived from user profiles that are used to give recommendations to the user. The rules are generated from the user profiles. The user profiles are created by clustering the user's web click streams. The main advantage of this approach is the very low cost when compared to collaborative recommendation systems; it can efficiently handle overlaps in users' interest and low main memory is required during recommendation time. Berners-Lee et al. [7] introduced semantic web. The semantic web is an extension of the World Wide Web. In semantic web the meaning of the web pages is well defined and structured in such a way that the computers and humans can work in cooperation. The semantic web creates an environment in which agents freely move from pages to pages and bring essential information to the users. Chau et al. [8] introduced a multiagent system named collaborative spider to support user collaboration in web mining. It supports collaboration by sharing complete search sessions based on postretrieval analysis. Dorigo and Sttzle [9] introduced ant colony optimization (ACO) where the ants coordinated actions and self-organizing principles are used to solve computational problems. ACO is inspired from the foraging behavior of ant colonies. Labroche et al. [10] introduced AntClust algorithm for grouping web usage sessions using chemical recognition systems in artificial ants. AntClust algorithm gains inspiration from ants' ability to differentiate between the nest mates and outsiders using the exchange of some chemicals. AntClust computes the similarity between the objects and groups the input web user sessions that represent the number of hits per page into clusters. Here, users with similar interest come in the same cluster. Kennedy and Eberhart [11] presented particle swarm optimization (PSO) which is an evolutionary swarm intelligence based computational model. PSO is inspired from bird flocks. Here, each swarm represents a solutions set. The swarms or particles fly through the solution space. Each position of the particle in the problem space represents a solution. At each move a fitness function is evaluated to identify closeness of particles solution to the global optimal solution. Here global best solution (*g*best) of the particle is the best solution found in the neighborhood and the personal best solution (*l*best) is the best position visited by the particle which until now are used to find the particles new position. Moawad et al. [12] introduced a new multiagent system based approach for personalizing the web search results. In this approach dynamic user profiles are created and maintained through implicit user feedback system. Saka and Nasraoui [13] introduced flocks of agent based recommendation system (Flock-Recom) to give recommendations to user for web personalization. It gains inspiration from the collaborative behavior of flocks of birds. Each agent represents a user. Agents are allowed to freely move in the visualization panel. Agents iteratively adjust the velocity and position on the visualization panel. Based on the neighboring agents on the visualization panel top-*n* recommendations are given to the user.

## 3. Background

### 3.1. Web Usage Mining

Web usage mining is the process of applying data mining techniques to web log data to discover interesting usage patterns [14]. It consists of the following steps:

1. Preprocessing the web log files.
2. Pattern discovery using data mining techniques [14–17].
3. Postprocessing.
4. Tracking evolving user profiles [18].

#### 3.1.1. Preprocessing the Web Log Files

Each entry in the web log files consists of IP address, URL viewed, and access time. The web log files extracted from the web server contain a huge amount of information. All these pieces of information are not needed for further processing. The quality of the patterns discovered after web usage mining process depends on how well you perform data cleaning and user session identification. Data cleaning includes filtering the crawler's request, request to graphics, and identifying unique sessions. The user session identification includes identifying the pages referenced by a user during a single visit to a site.

#### 3.1.2. Pattern Discovery Using Data Mining Techniques

Once the user sessions are identified, various data mining methods such as frequent item sets, clustering, classification, association rule mining, path analysis, neural network approaches, and heuristic approach methods can be applied to extract useful patterns from web log files. These discovered patterns identify users' interests, behavior, habits, and changes in their interest. A website can be personalized or customized for a user based these pieces of information, thereby increasing the profit of an organization.

#### 3.1.3. Postprocessing and Tracking Evolving User Profiles

User session categories [18] are summarized into user profiles. Tracking evolving user profiles includes comparing the user profiles generated in different months. This helps in identifying new groups of user profiles and merging or splitting of user profiles and inactive user profiles. All these changes in user profiles represent the changes in customer's interests or behaviors.

### 3.2. Swarm Intelligence

Swarm intelligence gains inspiration from several communities in nature such as fish schools, ant colonies, honey bees, and bird flocks. Swarm intelligence uses intelligent agents to handle copious information. An agent perceives the environment through sensors and it acts on the environment through actuators [19]. Intelligent agents can continuously perceive the dynamic conditions in the environment; it can perform actions to affect the conditions in the environment and performs reasoning to interpret perceptions. The flexibility of the software agents makes it possible to dynamically choose which actions to perform and their sequence in response to the state of its external environment.

### 3.3. Stimulated Annealing

Stimulated annealing [20] provides an optimal solution for the nearest neighbor search. Annealing is the process of heating a metal to its melting point and then cooling it back into solid state. Final structure of the metal depends upon cooling function. Slow cooling results in large crystals with low energy whereas fast cooling results in high energy state resulting in imperfections. Slow cooling always gives a better result.

## 4. The Proposed System

In the past, many research works were done in swarm intelligence for web usage mining like AntClust [10], particle swarm optimization (PSO) [11], Fab [4], collaborative filtering (CF) [1], and so forth. All these methods cannot model the dynamic behavior of users efficiently and the recommendations given to users lack ability to handle seasonality in users' interest. Because of the information overload problem in web usage data in many traditional swarm intelligence methods such as ACO and PSO, the number of iterations needed for the system before convergence is high.

In the present work, we propose a WebBluegillRecom-annealing dynamic recommender system. It uses the simulated annealing and swarm intelligence for identifying the interesting items to be recommended for the users. The WebBluegillRecom-annealing algorithm gains inspiration from the foraging behavior of bluegill fish. Swarm intelligence uses intelligent agents to handle abundant information on the web, thereby increasing scalability. Here, intelligent software agents are used to model artificial life. Intelligent software agents can handle the dynamic nature of online usage, thereby overcoming information overload problem. This flexibility property permits the artificial bluegill fish to model foraging behavior of real bluegill fish in different densities of prey in water. The learning capability of software agents allows continuous monitoring of users dynamic behaviors and gives predictions.

**Figure 1:**
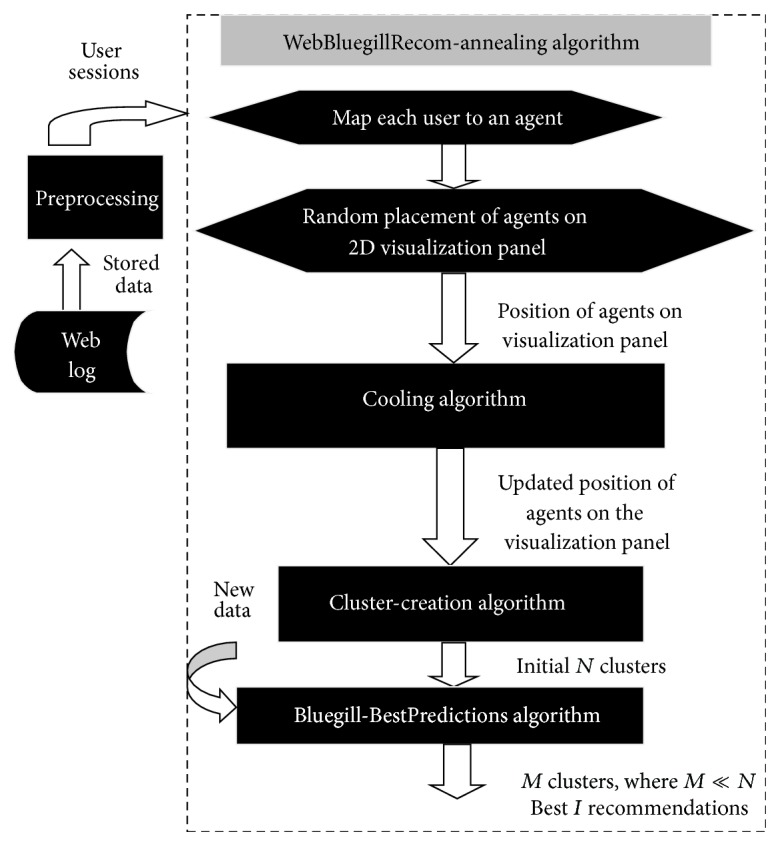
Steps involved in WebBluegillRecom-annealing dynamic recommender system.

The proposed WebBluegillRecom-annealing algorithm uses a cooling schema to make all agents in stable state. The cooling algorithm has been developed based on the simulated annealing approach. The cooling schema in the proposed WebBluegillRecom-annealing algorithm reduces the number of iterations required for the agents to enter into a stable low energy state.  Figure 1 shows the steps involved in the proposed dynamic recommender system.

In the proposed WebBluegillRecom-annealing dynamic recommender system initially each user obtained after the data cleaning process is mapped to an agent. The agents are placed randomly on the 2D visualization panel. A cooling algorithm is then applied to bring similar agents nearer to each other in the visualization panel. This gives an initial neighborhood for agents. A better neighborhood is formed in each iteration of the algorithm by iteratively adjusting the position of the agents on the visualization panel. That is, the users that exhibit similar behavior will form a hinterland. In order to handle dynamic data that is collected incessantly and to improve the quality of neighborhood, a dynamic clustering technique is applied. Recommendations are given to users as best *I* items preferred by the user's latest neighborhood.

The proposed WebBluegillRecom-annealing recommender system can handle the following challenges of web usage mining such as information overload problem, dynamic behavior of users, large number of iterations before convergence, and scalability and overspecialization in recommendations problem.

### 4.1. Preprocessing of Web Logs to Extract Input User Sessions

Web server log files are preprocessed and input user sessions are identified. Here the *i*th user session is encoded as an *A*-dimensional binary attribute vector [21] User_*i*_ with the following property:

(1) $$\begin{array}{c} {User}_{i}=\left\{\begin{array}{cc} 1, & \text{if user }i\text{ accessed URL }j,\\ 0, & \text{otherwise}. \end{array}\right. \end{array}$$

### 4.2. User Profile Creation Based on Data Mining Techniques

In this paper, a dynamic clustering based data mining technique is used to discover interesting online usage patterns. Unlike conventional clustering, in dynamic clustering [22], the whole input data need not be made available initially. The input data is collected continuously over time. Dynamic clustering technique has the ability to manage incoming dynamic data that represents the dynamic behaviors of users. Dynamic behaviors of users are due to the changes in user's interest or behavior or due to the addition or deletion of a web page. These discovered patterns can be used for the creation of user profiles and for giving recommendation to users.

#### 4.2.1. The Proposed WebBluegillRecom-Annealing Algorithm

In the proposed WebBluegillRecom-annealing algorithm (Algorithm 1), each user is mapped to an agent. All the agents are placed on the visualization panel randomly. To bring similar agents closer and dissimilar agents far apart, a cooling algorithm (Algorithm 2) is applied. Then the clusters of agents are formed using cluster-creation algorithm (Algorithm 3). It groups similar agents into the same cluster. That is, users having similar interests belong to the same cluster. This initial set of clusters can be used for further processing. These initial clusters are given as input to the Bluegill-BestPredictions algorithm (Algorithm 4). The Bluegill-BestPredictions algorithm can optimize these initial clusters by identifying a better neighborhood for agents in each cluster forming another hinterland. Moreover, it can assign new dynamic data representing a new dynamic behavior of user to the most similar cluster. It performs dynamic clustering of dynamic data and gives the users the finest recommendations by predicting the best *I* items preferred by the neighborhood agents. Bluegill-BestPredictions algorithm gives dynamic recommendations to users. Since the recommendations are dynamic, the WebBluegillRecom-annealing algorithm can satisfy the needs of old and new users. The following part explains each of these algorithms in detail.

In the proposed WebBluegillRecom-annealing algorithm, initially each user is mapped to an agent. Agents are placed on the visualization panel randomly. Visualization panel is a two-dimensional plane represented by *x*-*y* coordinates. The *x*-axis and *y*-axis values range from 0 to 1.

To bring similar agents closer and dissimilar agents far apart in the visualization panel, we use a cooling algorithm based on annealing concept used in metals. The attributes of this thermodynamic simulation can be mapped into stimulated annealing optimization, where a system state represents feasible solutions, energy represents costs, change of state represents the neighboring function, temperature represents control parameter, and frozen state represents final solution. Initially when the temperature is high, it accepts bad moves. This is because starting solution may not be too good because of the difficulty of escaping from neighborhood. But when the temperature is low, it almost rejects bad moves. The best ever result is kept as the final solution.

The inputs to the cooling algorithm (Algorithm 2) are agents, their position on the visualization panel, and the temperature length. In Step (1) of Algorithm 2 an initial solution Ω_0_ is generated randomly and assign this as the final solution Ω^*∗*^. In Step (2), initial temperature value is generated. In line 5, a new solution is created by selecting neighboring agent that is similar to the solution Ω^*∗*^. In this algorithm a cost function is calculated using cosine similarity of agents. Here, the cost function is to maximize the cosine similarity (CS) of agents. Cosine similarity between any two agents represents the similarity between users that are mapped to that agent. Cosine similarity can handle qualitative and quantitative data. It can also handle high dimensional sparse data. In Step (6), the change in the cost function is calculated. In line 7, it checks whether the change in cost function (energy) is decreased. If energy is decreased then the new state is accepted (line 8). Otherwise, the new state with probability *e* ^∧^(Δ/*T*) (line 10) is accepted. In line 14, a geometric temperature reduction is used, where 0.8 ≤ *r* ≤ 0.99. To get good results *T* should be adjusted in such a way with small number of iterations at higher temperature and larger number of iterations at low temperature. For the final solution to be independent of the starting one the initial temperature should be high enough. When the temperature is low there are no uphill moves. When a given minimum value of temperature is reached or when a certain amount of looping has been performed without accepting a new solution, the algorithm is stopped (line 14).

The cooling algorithm applied is a greedy heuristic allowing the agents to move from current positions to the best neighboring solution. The cooling algorithm returns the agents with their new position on the visualization panel. Now similar agents lie close in the visualization panel. As the distances between the agents on the visualization panel increase their similarity decreases. To avoid local minima, it supports uphill moves. After applying the cooling function, agents converge to a frozen low energy state where similar agents are located nearer to each other. The usage of cooling algorithm reduces the number of iterations. These agents with their updated positions on the visualization panel after applying cooling algorithm are given as input to the cluster-creation algorithm.

In cluster-creation algorithm (Algorithm 3) Distance (*A*_*i*_, *A*_*j*_) represents the distance between agents *A*_*i*_ and *A*_*j*_ on the visualization panel. *σ*_*k*_^2^ represents the mean squared error or average dissimilarity between the cluster prototype and the data records [21]. Mean squared error is calculated using

(2) $$\begin{array}{c} {\sigma_{k}}^{2}=\frac{\sum_{S^{\left(i\right)}£\chi_{k}}d_{ki}^{2}}{\left|\chi_{k}\right|}, \end{array}$$

where *S* ^(*i*)^ represents the *i*th user session and *χ*_*k*_ represents the set of sessions assigned to *k*th cluster. *d*_*ki*_ is the distance from *S* ^(*i*)^ to *χ*_*k*_. Initial clusters of agents are formed by grouping the agents that lie within a distance threshold and whose mean squared error lies within *σ*_th_^2^ into a cluster (lines 4 to 6). Thus the agents within a cluster represent similar users. These clusters are given as input to the Bluegill-BestPredictions algorithm.

In the Bluegill-BestPredictions algorithm (Algorithm 4) the foraging behavior of bluegill fish is used to give dynamic recommendations to user. A bluegill sunfish [23] eats a prey to maximize its energy intake. At high density of prey, the bluegill fish eats a diet made up of larger prey. At a medium density of prey, bluegill fish eats large prey over a small prey. At this time bluegill fish becomes more selective. That is, instead of time spent in capturing and eating smaller ones, the bluegill fish can maximize its energy by eating larger ones. At a low density of prey the bluegill fish eats large, medium, and small prey as they are encountered, thus maximizing its energy intake. The behavior of bluegill fish is dynamic because the way in which it catches its prey is different in different densities of prey. The bluegill fish maximizes its energy in an optimized way, that is, more energy in less time. These behaviors of bluegill fish can be used to give dynamic recommendations to users. The proposed dynamic recommender system gives users better recommendations in less time, that is, lesser number of iterations, without compromising the quality in terms of coverage and precision. The usage of intelligent software agents helps us to simulate this artificial life. The intelligent agents are highly flexible to adapt to the dynamic behavior of users.

The Bluegill-BestPredictions algorithm, described as Algorithm 4, supports dynamic clustering of dynamic data items and provides dynamic predictions to users. For dynamic clustering [19], the complete data set need not be made available initially. The data is continuously collected over time. Whenever new data is added, it requires costly update of the clusters. The new web click streams may be due to change in user's interest or due to the addition or deletion of a web page from given website. Application of cooling algorithm and cluster formation algorithm results in the visualization of initial clusters in the visualization panel and similar agents belonging to the same cluster. Distance between the agents in the visualization panel represents the similarity between the users.

In line 2 of Algorithm 4, the centroid of each cluster obtained as the output of cluster-creation algorithm is mapped as a lake. In line 3, data_*a*_ represents the input dynamic data and is mapped to the corresponding agent in lines 4 and 6. The data_*a*_ is considered as the bluegill fish. Here each cluster is considered as a lake with different density of prey and the agents in each cluster (lake) are considered as the prey. In line 9, the similarity of the agent_*a*_ and Lake_*i*_ is compared using (3). Here, we use web session similarity [19, 21] between URLs as similarity measure based on the fact that an agent represents user sessions:

(3) Web  session  similarityveci,vecj=∑ki=1A∑kj=1AvecikivecjkjSuki,kj∑ki=1Aveciki∑kj=1Avecjkj,

where vec_*i*_ represents the user session vectors that are prepared in the preprocessing step and *S*_*u*_(*i*, *j*) represents the syntactic similarity between the *i*th and *j*th URLs. Consider

(4) $$\begin{array}{c} S_{u}\left(\;i,j\;\right)=\min\left(\;1,\frac{\left|p_{i}{\cap p}_{j}\right|}{\max\left(1,\max\left(\left|p_{i}\right|,\left|p_{j}\right|\right)-1\right)}\;\right). \end{array}$$

For a given URL Ui, *p*_*i*_ represents the path traversed from the root node which is the main page to the node corresponding to the *i*th URL. |*p*_*i*_| indicates the length of this path [19].

If the similarity of agent_*a*_ and Lake_*i*_ is greater than a predefined threshold (Maxth) value we assume that bluegill enters a lake containing high density of prey (line 9). In high density of prey, bluegill maximizes its energy by eating only large size prey. Here agent_*a*_ identifies the most similar neighboring agents (line 11) in that cluster until the stopping condition (line 18). The Eat algorithm (Algorithm 5) groups all these similar agents into a new cluster.

If the similarity of agent_*a*_ and Lake_*i*_ is less than the predefined minimum threshold (Minth) value we assume that bluegill enters a lake containing low density of prey (line 20). In low density of prey instead of waiting for larger prey bluegill maximizes its energy by eating any kind of prey as they get nearer. That is, it does not maintain any priority for diet. It will eat medium size prey or small size prey or large size prey as they come nearer to bluegill. If the similarity of agent_*a*_ and its neighboring agent in the visualization panel is an element of high_range (lines 22 to 25 assume bluegill eats large size prey) then the agent_*a*_ and neighboring agent are grouped together. Again the similarity between agent and the next neighbor agent is compared. If the similarity is an element of midrange we assume bluegill eats medium size prey (lines 33 to 36) or if the similarity is an element of low range we assume bluegill eats small size prey (lines 42 to 45). This process of eating is continued until bluegill gets sufficient energy for its survival.

If the similarity of agent_*a*_ and Lake_*i*_ is between Minth and Maxth we assume bluegill enters a lake containing medium density of prey (line 47). In this situation bluegill eats large and medium size preys. That is, instead of wasting time on capturing small size prey it prefers to capture larger and medium size prey. Lines 48 to 67 perform this behavior of bluegill fish. That is, here agent_*a*_ attracts high and medium similar agents.

These movements of agents result in a new group of agents or merging or splitting of some clusters or even deletion of some clusters. The agents with their new positions are visualized in visualization panel (line 71). In line 73, all the URLs that are visited more than a predefined threshold value Url_count_threshold are displayed for each valid cluster. Line 77 assigns the set of neighboring agents of agent_j_ that lies within a distance *d*best to *G*(*a*). Line 78 assigns the frequently preferred items by all the agents in *G*(*x*) to *F*(*G*(*a*)). In line 79, the most frequent *I* items in the set *F*(*G*(*x*)) are given as recommendations to user *u* represented by the agent agent_j_. This kind of recommendations overcomes the overspecialization problem in many traditional recommendation systems. The recommendations given by the proposed recommender system include variety in recommendations. That is, here recommendations are given not only based on that user profile of users but also based on the preferences of neighboring users. These recommendations can be used for personalizing a website, thereby improving customer relationship management (CRM).

Here, the performance of the proposed WebBluegillRecom-annealing algorithm is compared with the traditional collaborative filtering based recommender system. The performances are evaluated in terms of coverage, precision, and *F*1 measure. The experimental results show that the proposed WebBluegillRecom-annealing dynamic recommender system performs better recommendations than the traditional collaborative systems.

## 5. Experimental Results

In Section 5 we start with the 2D visualization of agents on the visualization panel. Then we proceed with the visualization of clusters of agents obtained by the cluster-creation algorithm and Bluegill-BestPredictions algorithm. Then we show inter- and intracluster similarity measures of the obtained clusters. Then we proceed with the recommendations given to the users. And finally we compare the quality of the proposed dynamic recommender system with the traditional collaborative filtering techniques.

**Figure 2:**
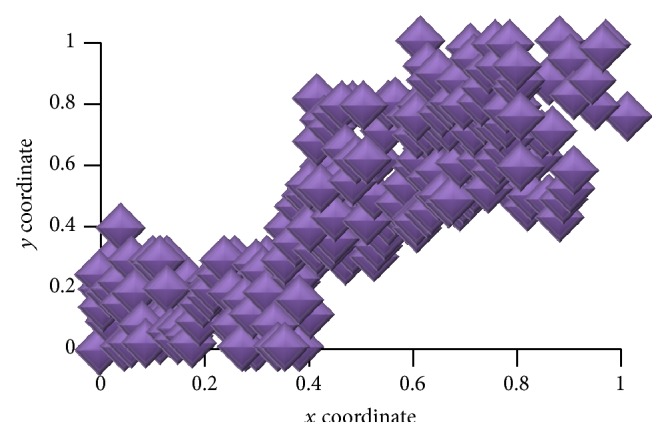
Initial position of agents.

The proposed WebBluegillRecom-annealing dynamic recommender system is implemented on high dimensional real life data example. It is implemented using Java agent development environment (JADE).  Figure 2 shows the random placement of agents in the visualization panel.

**Figure 3:**
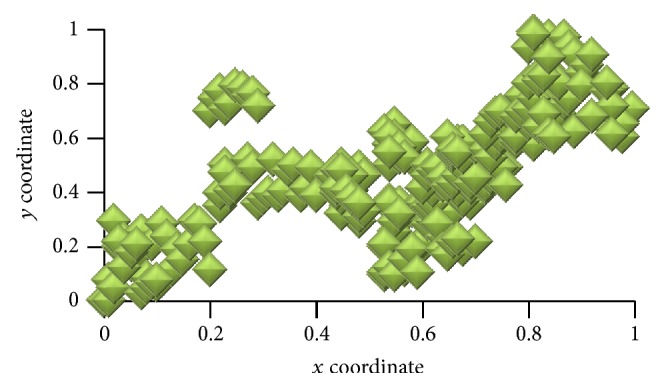
New position of agents after applying cooling algorithm.

In Figure 2, visualization panel is a two-dimensional plane represented by *x*-*y* coordinates. The *x*-axis and *y*-axis values range from 0 to 1.  Figure 3 shows the position of agents on the visualization panel after applying cooling algorithm.

**Figure 4:**
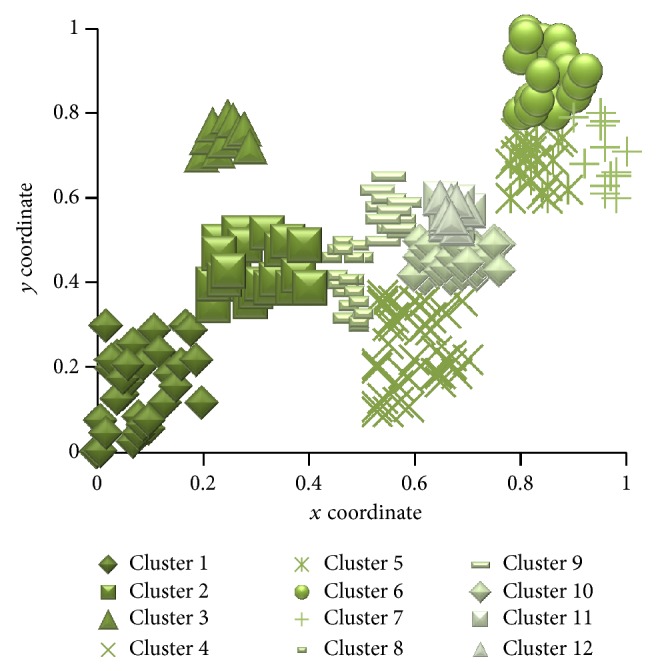
Clusters of agents obtained after applying cluster-creation algorithm.

In Figure 3, similar agents lie nearer in the visualization panel. To get better neighboring agents, a slow cooling method is adopted. That is, the value of *T* is reduced slowly. Here a geometric temperature reduction is used. Here *r* = 0.96 (line 14 of Algorithm 2) and TL = 0.005. Values are chosen by trial and error.  Figure 4 shows the clusters of agents after applying cluster-creation algorithm.

Here the neighboring agents that lie within a distance threshold *d*_th_ = 0.15 and when the average dissimilarity between the clusters and their member agents is less than *σ*_*k*_^2^ = 0.006 are moved into the same cluster. Values are set by trial and error. Agents in the same clusters represent the users with similar behavior. The initial clusters are given as input to the Bluegill-BestPredictions algorithm.

**Figure 5:**
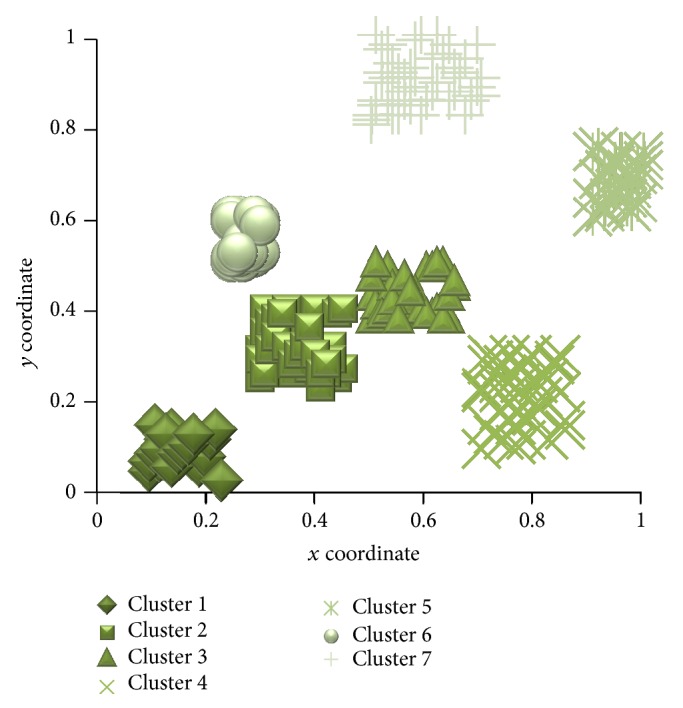
Clusters of agents obtained after applying Bluegill-BestPredictions algorithm.

Figure 5 shows the clusters of agents obtained after applying Bluegill-BestPredictions algorithm. In the Bluegill-BestPredictions algorithm for higher density cluster the Maxth value ranges from 0.75 to 1.0. For lower density cluster we set the Minth value as 0.40. In medium density cluster the similarity lies between 0.40 and 0.75. In high density cluster high_range means similarity above 75% of Maxth. In lower density cluster, high_range means similarity above 75% of Minth, mid_range means similarity between 45% and 75% of Minth, and low_range means similarity below 45% of Minth. In medium density cluster high_range means similarity above 75% of obtained medium density cluster similarity value. Here, mid_range lies within 45% and 75% of medium density cluster similarity value. These parameters are set by trial and error method. In Figure 5 Clus_threshold = 10 and Url_count  _threshold = ICTF*∗*Clus_threshold [19], where ICTF denotes item count threshold frequency [19] that represents the minimum number of URL patterns that represent that session. Here ICTF = 0.10. An ICTF value is a real number and it lies between 0 and 1. Clus_threshold represents the minimum cluster size required for a valid cluster.

Quality of the obtained clusters can be measured in terms of intracluster similarity and intercluster similarity. For better clusters intracluster similarity value should always be higher than intercluster similarity value. Intracluster similarity value represents the similarity between the elements of that cluster. Intercluster similarity represents the similarity between the elements of a cluster with the members of the other cluster.  Table 1 represents intracluster similarity and intercluster similarity of obtained clusters using WebBluegillRecom-annealing algorithm.

From Table 1 we can observe that better clusters are obtained by optimizing the clusters generated by cluster-creation algorithm by the Bluegill-BestPredictions algorithm. From this table we can notice that whenever intracluster similarity increases intercluster similarity decreases. This is a sign of good quality clusters.  Table 2 shows the sample user profiles generated by WebBluegillRecom-annealing algorithm.

Table 2 shows some user profiles in cluster 6 representing a user's interest after applying WebBluegillRecom-annealing algorithm. WebBluegillRecom-annealing system gives the best recommendations to a user with *d*best = 0.04 (line 68 of Algorithm 4). Next time mia logs onto the website the following recommendations are given in Table 3.

Table 3 shows that recommendations are given to a user not only based on that user's preference but also based on the other users' (or agents) items of interest that lie within the distance *d*best in the same cluster. Here the most frequent *I* = 6 items are selected. The recommendations made by the WebBluegillRecom-annealing dynamic recommender system contain variety. That is, the recommendations given to a user are not limited to that user's interest but also include the items more frequently liked by other users. The goodness of these recommendations is evaluated in terms of coverage and precision [18]. Here precision represents a summary profile's items which are all correct or included in the original input data; that is, they include only the true data items:

(5) $$\begin{array}{c} Prec_{ij}=\frac{s_{j}\cap p_{i}}{p_{i}}. \end{array}$$

Coverage represents summary profile's items which are complete compared to the data that is summarized; that is, they include all the data items:

(6) $$\begin{array}{c} Cov_{ij}=\frac{s_{j}\cap p_{i}}{s_{j}}. \end{array}$$

Here *s*_*j*_ is a summary of input sessions and *p*_*i*_ represents discovered mass profile. A precision value of 1 indicates that every recommended item is an element of the original truth set. A coverage value of 1 indicates that all items in the original truth set are recommended. Efficiency recommender system depends on how well it can balance the precision and coverage *c*. To calculate coverage and precision the generated user profiles and the input user sessions are used. Here the precision, coverage, *F*1 measure, and variety in recommendations of the proposed WebBluegillRecom-annealing dynamic recommender system are compared with the traditional collaborative filtering system. Traditional CF approach is selected as baseline to evaluate the performance of the proposed recommender system because both our approach and CF approach use nearest neighbor property. In CF approach if a user *n* is similar to user *u*, then *u* is considered as the neighbor of *n*. In CF approach to generate a prediction for an item *i* it analyzes the ratings for *i* from users in *u*'s neighborhood using Pearson correlation [1].

**Figure 6:**
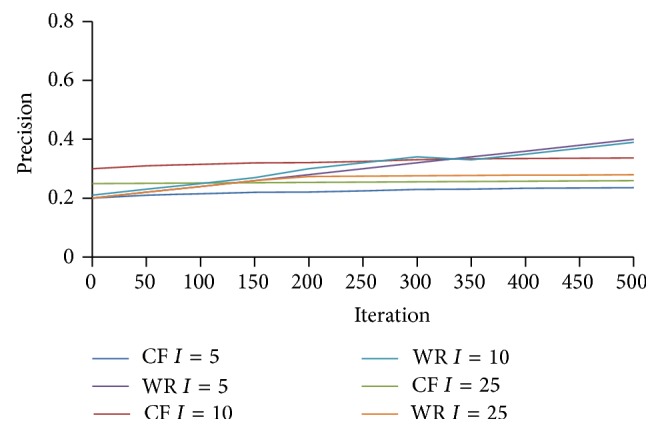
WebBluegillRecom-annealing system (WR) is compared to standard collaborative filtering (CF) system on precision, 248 users.

In Figure 6 the proposed WebBluegillRecom-annealing dynamic recommender system is compared with the traditional collaborative filtering system on precision for different *I* values.

**Figure 7:**
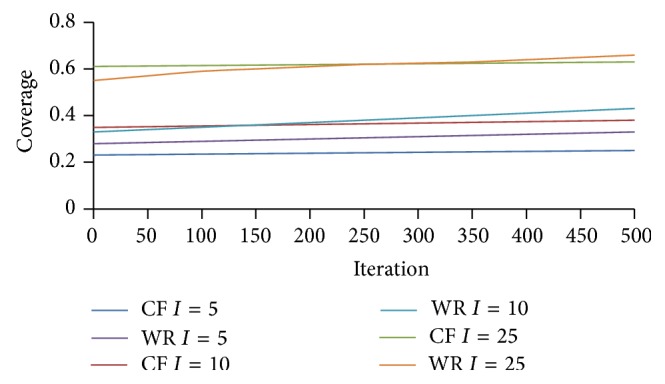
WebBluegillRecom-annealing system (WR) is compared to standard collaborative filtering (CF) system on coverage, 248 users.

From Figure 6 we can observe that the precision values for WebBluegillRecom-annealing dynamic recommender system are slightly better than the collaborative filtering systems for small values of *I*. Better recommendations are obtained when *I* = 5. This indicates that the recommendations given by WebBluegillRecom-annealing system are an element of the original input datasets when compared to the collaborative filtering system. In Figure 7 the proposed WebBluegillRecom-annealing dynamic recommender system is compared with the traditional collaborative filtering system on coverage.

From Figure 7 we can observe that, as *I* increases, the coverage values also increase for WebBluegillRecom-annealing dynamic recommender system and collaborative filtering systems. Moreover, for a particular value of *I*, as the iteration increases, there is an improvement in coverage values for WebBluegillRecom-annealing dynamic recommender system compared with the collaborative filtering system. WebBluegillRecom-annealing dynamic recommender system has much better values for coverage than the collaborative filtering system for small values of *I*. But for higher values of *I* there is only a slight improvement in coverage value compared to the collaborative filtering system. The recommendations given by the WebBluegillRecom-annealing system contain more elements in the original input datasets than the collaborative filtering system.

**Figure 8:**
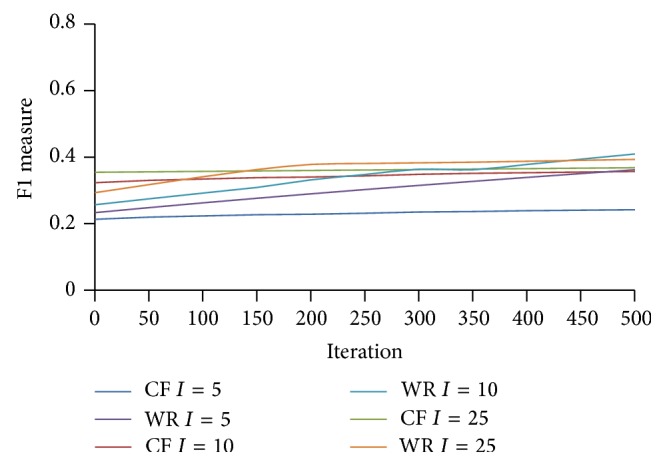
WebBluegillRecom-annealing system (WR) is compared to standard collaborative filtering (CF) system on *F*1 measure, 248 users.

Balancing of precision and coverage can be represented using *F*1 measure [19]. Higher values for *F*1 measure represent more balanced coverage and precision. In Figure 8 the proposed WebBluegillRecom-annealing system is compared with the traditional collaborative filtering system on *F*1 measure:

(7) $$\begin{array}{c} F1,ij=\frac{2\left(cov_{ij}*prec_{ij}\right)}{cov_{ij}+prec_{ij}}. \end{array}$$

From Figure 8 we may observe that the *F*1 measure is higher for WebBluegillRecom-annealing system when compared to collaborative filtering system. That is, better balancing of coverage and precision is possible in WebBluegillRecom-annealing system when compared to collaborative filtering systems. In the WebBluegillRecom-annealing system the same items are not recommended to users again and again. It includes variety in recommendations.

**Figure 9:**
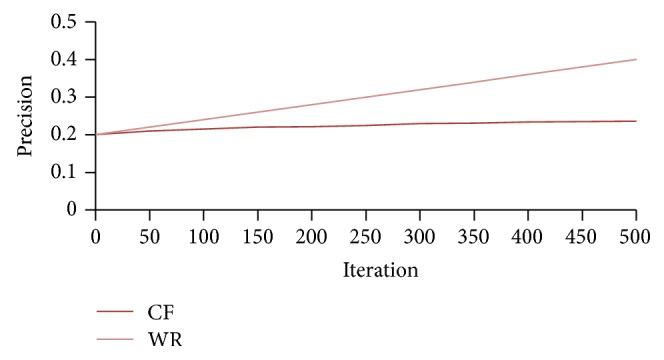
WebBluegillRecom-annealing system (WR) is compared to standard collaborative filtering (CF) system on precision, *I* = 5, 248 users.

Figure 9 shows the comparison of the WebBluegillRecom-annealing system with traditional CF systems on precision when *I* = 5.

**Figure 10:**
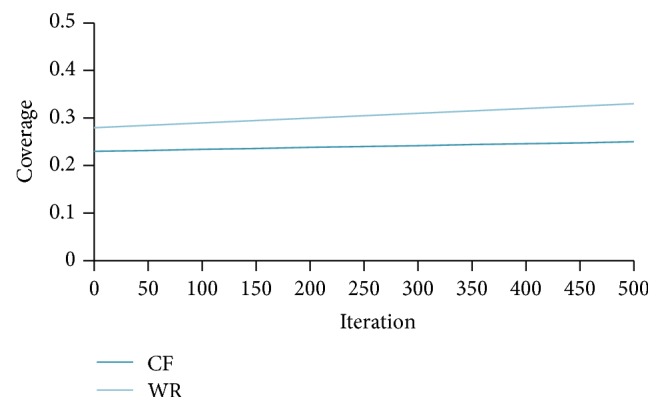
WebBluegillRecom-annealing system (WR) is compared to standard collaborative filtering (CF) system on coverage, *I* = 5, 248 users.

From Figure 9 we can observe that the proposed recommender system can face the challenge of reducing the number of iterations required in comparison with traditional CF. For the proposed WebBluegillRecom-annealing system when the number of iterations increases, the precision also increases. At the same time for a particular number of iterations the precision provided by the proposed method is better than the precision achieved for the same number of iterations in CF systems. That is, the required precision could be achieved with less number of iterations required for CF method. The annealing approach used in the algorithm reduces the number of iterations actually required to achieve a particular precision. To attain this precision traditional CF needs a higher number of iterations.  Figure 10 shows the comparison of the WebBluegillRecom-annealing system with traditional CF systems on coverage when *I* = 5.

**Figure 11:**
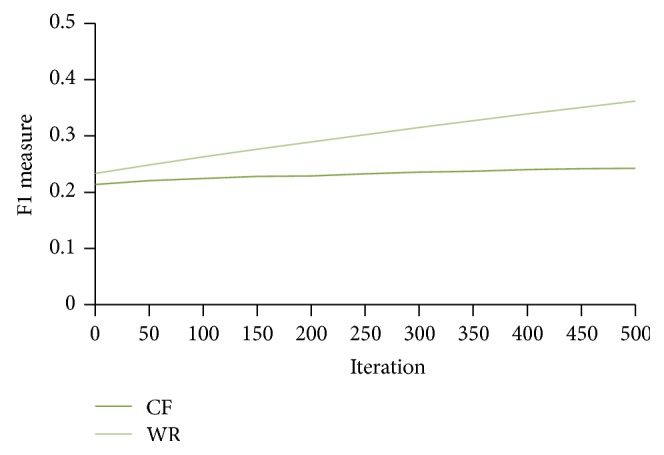
WebBluegillRecom-annealing system (WR) is compared to standard collaborative filtering (CF) system on *F*1 measure, *I* = 5, 248 users.

From Figure 10 we can observe that, for a particular number of iterations, the coverage provided by the proposed method is better than the coverage achieved for the same number of iterations in CF systems.  Figure 11 shows the comparison of the WebBluegillRecom-annealing system with traditional CF systems on *F*1 measure when *I* = 5.

**Figure 12:**
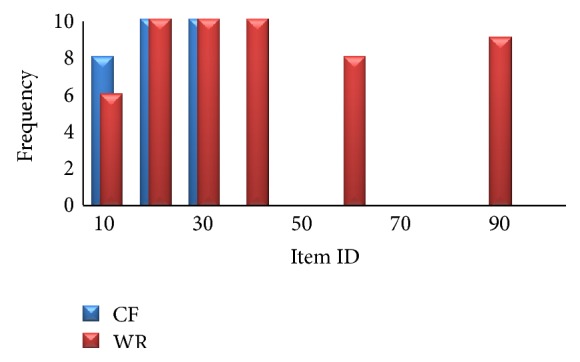
WebBluegillRecom-annealing system (WR) is compared to standard collaborative filtering (CF) system on variety averaged over 10 different runs per 1 active user, for *I* = 5 recommendations, at iteration 100, 248 users.

From Figure 11 we can observe that the proposed method gives better *F*1 measure when compared to traditional CF system in less number of iterations. That is, to attain a particular *F*1 measure the traditional CF system needs more iterations.  Figure 12 shows the number of times each item is recommended for a specific user over 10 different runs.

Variety means number of distinct recommended items [19]. In Figure 12, *x*-axis represents the item ID and *y*-axis represents the frequency of recommendations for a particular item. From Figure 12 we can observe that while traditional CF systems kept recommending the same items, WebBluegillRecom-annealing system adds variety without losing coverage, precision, and *F*1 measure.

To summarize, Figures 6 to 12 show that, in the WebBluegillRecom-annealing system, as the iteration increases, there is an increase in similarity of neighbors resulting in better recommendations. As the number of iterations increases, better recommendations are given by the WebBluegillRecom-annealing dynamic recommender system than the collaborative filtering system. WebBluegillRecom-annealing dynamic recommender system gives varieties in recommendations, thereby overcoming overspecialization in recommendations. Results of returning items that are too similar to the previously rated items by the user are called overspecialization. The proposed Bluegill-BestPredictionsalgorithm used in the proposed recommender system gives dynamic recommendations each time to a user. That is, it recommends a variety of new items for the user. Hence, in the proposed system, recommendations are given in accordance with the users' varying interests. These improvements are due to the dynamic nature of foraging behavior of agents that forms dynamic neighborhood. More variety in recommendations helps the customers to review a larger number of items before buying. These interactions encourage the customers to visit the web page more frequently, thus improving customer relationship management. Since the proposed method gives wide variety of recommendations in comparison with the traditional CF method, it lures or encourages the customers to visit more frequently the website, enriching the relationship with the customer. Since a larger number of recommendations are given, customer interacts with the website more frequently. This may drive sales growth too. In turn, it requires more information to be provided by the business community regarding the product to the customers. That is, it improves customer relationship management (CRM).

**Figure 13:**
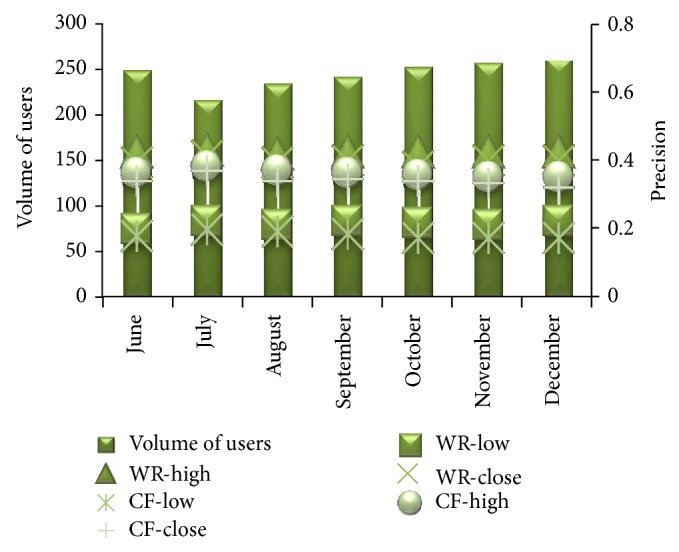
WebBluegillRecom-annealing system (WR) is compared to standard collaborative filtering (CF) system on precision for different volume of users in different months.

Figure 13 shows the comparison of the proposed WebBluegillRecom-annealing system and collaborative filtering system on precision for six months on varying number of users. It is based on the results of recommendations given to users. In Figure 13 the primary horizontal axis represents the months, the primary vertical axis represents the number of users that access the website in each month, and the secondary vertical axis represents the precision of recommendations given to users. WR-low and CR-low represent the lowest value of precision obtained in a month by evaluating the recommendations given to users in that month by the WebBluegillRecom-annealing dynamic recommender system and the collaborative filtering system, respectively. WR-high and CR-high represent the highest value of precision obtained in a month by evaluating the recommendations given to users in that month by the WebBluegillRecom-annealing dynamic recommender system and the collaborative filtering system, respectively. WR-close and CF-close represent the average value of precision obtained in a month by evaluating the recommendations given to users in that month by the WebBluegillRecom-annealing system and the collaborative filtering system, respectively.

**Figure 14:**
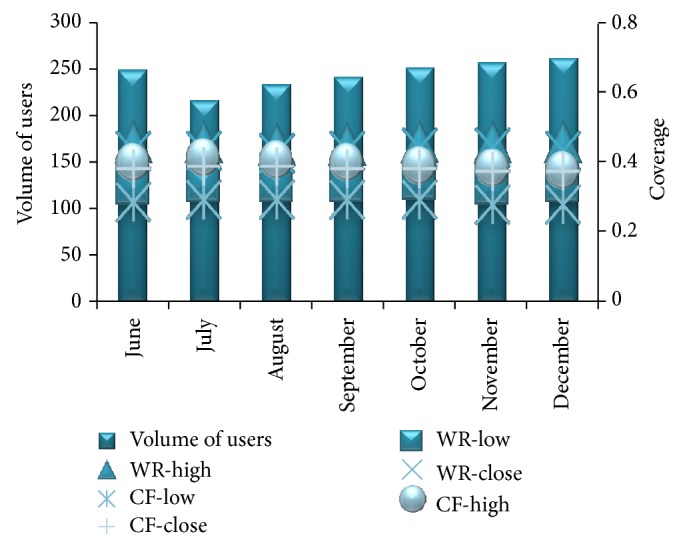
WebBluegillRecom-annealing dynamic recommender system (WR) is compared to standard collaborative filtering (CF) system on coverage for different volume of users in different months.

From Figure 13 we can observe that the WebBluegillRecom-annealing system has higher values of precision when compared to collaborative filtering system. In WebBluegillRecom-annealing system the precision is not that much affected by the number of users. But in the collaborative system there is a reduction in precision value with the increase in the number of users. That is, scalability is better supported by the proposed WebBluegillRecom-annealing system.  Figure 14 represents the comparison of WebBluegillRecom-annealing system with the standard collaborative filtering system on coverage for different number of users in different months.

**Figure 15:**
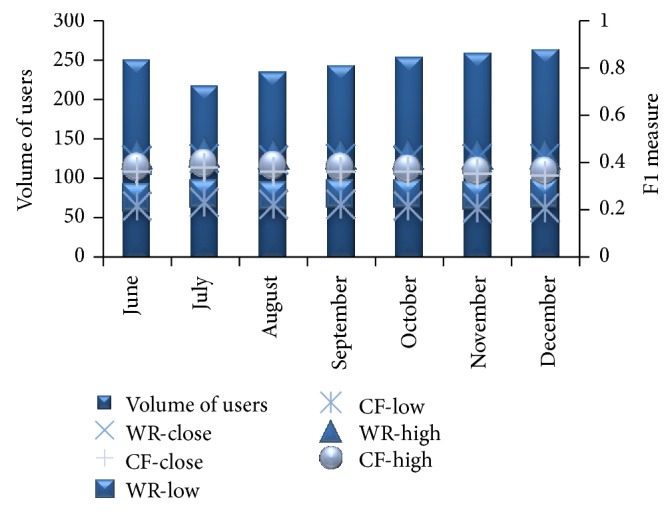
WebBluegillRecom-annealing system (WR) is compared to standard collaborative filtering (CF) system on coverage for different volume of users in different months.

In Figure 14, WR-high and CR-high represent the highest value of coverage obtained in a month by evaluating the recommendations given to users in that month by the WebBluegillRecom-annealing dynamic recommender system and the collaborative filtering system, respectively. WR-close and CF-close represent the average value of coverage obtained in a month by evaluating the recommendations given to users in that month by the WebBluegillRecom-annealing dynamic recommender system and the collaborative filtering system, respectively. WR-low and CR-low represent the lowest value of precision obtained in a month by evaluating the recommendations given to users in that month by the WebBluegillRecom-annealing system and the collaborative filtering system, respectively. From Figure 14 we can observe that the coverage is higher for WebBluegillRecom-annealing dynamic recommender system. In the WebBluegillRecom-annealing system coverage of the recommendations is not that much affected by the increase in the number of users when compared to collaborative filtering systems.  Figure 15 shows the comparison of the proposed recommender system and collaborative filtering system on *F*1 measure for six months.

In Figure 15 we can observe that *F*1 measure is higher for the proposed WebBluegillRecom-annealing dynamic recommender algorithm. We can also observe that better balancing coverage and precision are possible even though the number of users is increased.

To summarize, Figures 13, 14, and 15 show that WebBluegillRecom-annealing dynamic recommender system has better scalability than the traditional collaborative filtering systems. The “low” value may be due to a sudden rare move by the user. Even in these rare moves WebBluegillRecom-annealing dynamic recommender system has slightly better values for precision, coverage, and *F*1 measure than the collaborative filtering systems. WebBluegillRecom-annealing dynamic recommender system includes variety in recommendations without compromising quality in terms of coverage, precision, *F*1 measure, and scalability. These improvements are due to the dynamic nature of foraging behavior of bluegill agents which attracts (eats) similar agents to it resulting in a better neighborhood. These recommendations can be used for personalizing or customizing a website, thereby increasing customer relationship management.

## 6. Conclusion

In this paper a new dynamic recommender system called WebBluegillRecom-annealing system is presented. The proposed system is based on the swarm intelligence that gains inspiration from the dynamic foraging behavior of bluegill fish. The artificial life is simulated using software agents. WebBluegillRecom-annealing dynamic recommender system is capable of handling dynamic data. It uses an annealing approach to identify the initial best neighborhood for agents, thereby reducing the number of iterations before convergence. The WebBluegillRecom-annealing recommender system includes variety in recommendations, thereby overcoming the overspecialization problem in some traditional recommendation systems. The results obtained are compared with the traditional collaborative filtering system. The experimental results show that the WebBluegillRecom-annealing recommender system can better handle dynamic behavior and seasonality in users' interest than the traditional collaborative filtering systems. The experimental results show that the recommendations given by WebBluegillRecom-annealing system have better values for precision, coverage, and *F*1 measure than the collaborative filtering system. The proposed dynamic recommender system reduces the number of iterations before convergence when compared to traditional CF recommender systems. Moreover, in WebBluegillRecom-annealing system the quality of recommendations is not much affected by the increase in the number of users. That is, WebBluegillRecom-annealing system has improved scalability compared with the traditional collaborative filtering system. The recommendations given by the WebBluegillRecom-annealing system can be used for customizing a website, thereby improving customer relationship management (CRM). The main limitation of this method is that several parameters have to be set a priori. In the future this work can be extended to track evolving user profiles.

## Figures and Tables

**Algorithm 1:**
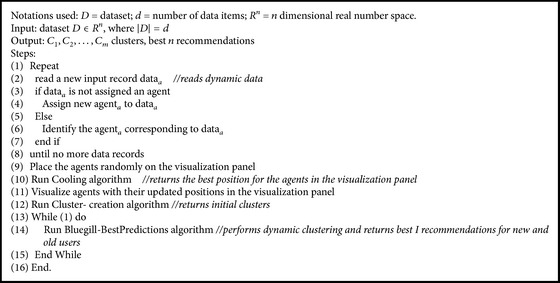
WebBluegillRecom-annealing algorithm.

**Algorithm 2:**
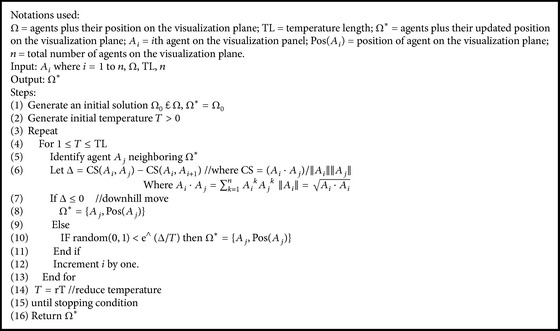
Cooling algorithm.

**Algorithm 3:**
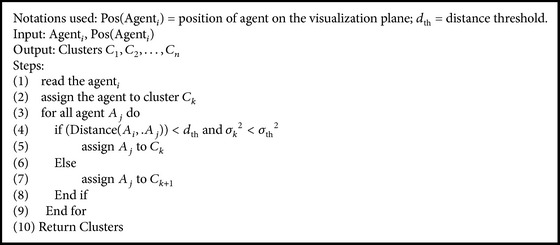
Cluster-creation algorithm.

**Algorithm 4:**
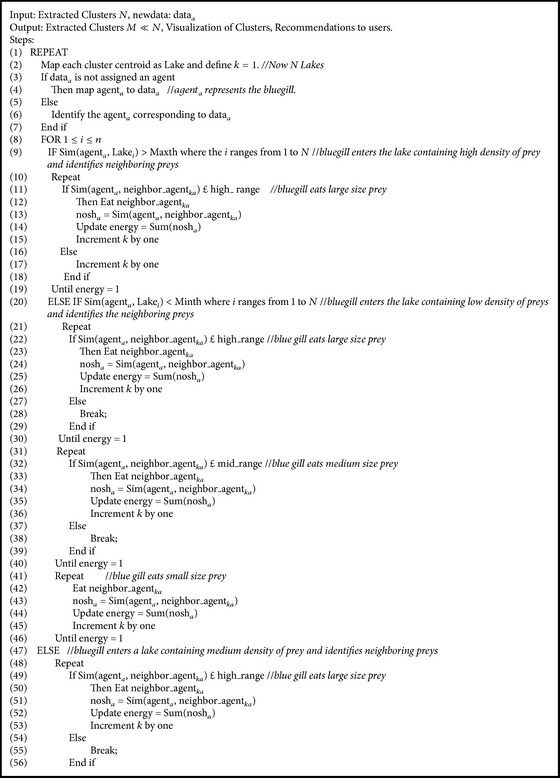
Bluegill-BestPredictions algorithm.

**Algorithm 5:**
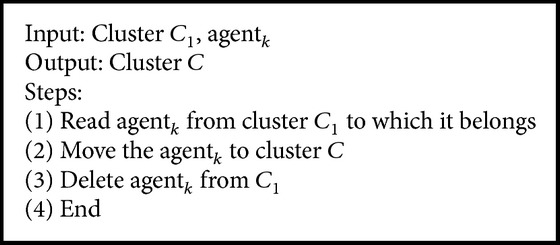
Eat algorithm.

**Table 1:** Average results of 10 runs of WebBluegillRecom-annealing algorithm with ICTF = 0.1 and *Clus_threshold* = *10*.

Cluster-creation algorithm			Bluegill-BestPredictions algorithm	
Intracluster similarity	Intercluster similarity	Run	Intracluster similarity	Intercluster similarity
0.437 (0.014)	0.014 (0.001)	10	0.468 (0.011)	0.009 (0.003)
0.409 (0.019)	0.019 (0.003)	5	0.399 (0.019)	0.010 (0.001)

**Table 2:** Sample user profiles generated by WebBluegillRecom-annealing algorithm with ICTF = 0.1 and *Clus_threshold* = *10. *

URL frequency	URL
Profile 1	
0.4	roshni/courses/cse309/notes.html
0.39	roshni/courses/cse309/slides.html
0.35	roshni/courses/cse312/notes.html

Profile 3	
0.42	mia/courses/cse312/slides.html
0.41	mia/courses/cse312/notes.html
0.39	mia/courses/cse312/assignments.html
0.24	mia/courses/cse312/
0.18	mia/courses/cse312/quiz.html
0.12	mia/courses/cse312/internals.html
0.11	mia/courses/cse312/proj

Profile 4	
0.41	neha/courses/cse475
0.37	neha/courses/cse312/
0.36	neha/courses/cse309/slides.html
0.24	neha/courses/cse475/notes.html

**Table 3:** Sample recommendations given to a user by the WebBluegillRecom-annealing dynamic recommender system with *I* = 6.

User → mia	courses/cse309/notes.html
	courses/cse309/slides.html
	/courses/cse312/slides.html
	/courses/cse312/notes.html
	/courses/cse312/assignments.html
	/courses/cse475
